# Deep learning based synthetic CT from cone beam CT generation for abdominal paediatric radiotherapy

**DOI:** 10.1088/1361-6560/acc921

**Published:** 2023-05-05

**Authors:** Adam Szmul, Sabrina Taylor, Pei Lim, Jessica Cantwell, Isabel Moreira, Ying Zhang, Derek D’Souza, Syed Moinuddin, Mark N. Gaze, Jennifer Gains, Catarina Veiga

**Affiliations:** 1 Centre for Medical Image Computing, Department of Medical Physics and Biomedical Engineering, University College London, London, United Kingdom; 2 Wellcome/EPSRC Centre for Interventional and Surgical Sciences, University College London, London, United Kingdom; 3 Department of Oncology, University College London Hospitals NHS Foundation Trust, London, United Kingdom; 4 Radiotherapy, University College London Hospitals NHS Foundation Trust, London, United Kingdom; 5 Department of Medical Physics and Biomedical Engineering, University College London, London, United Kingdom; 6 Radiotherapy Physics Services, University College London Hospitals NHS Foundation Trust, London, United Kingdom

**Keywords:** cone-beam computed tomography (CBCT), computer tomography (CT), cycle-consistent generative adversarial networks (cycleGANs), synthetic images, childhood cancer, abdominal neuroblastoma, paediatric radiotherapy

## Abstract

*Objective*. Adaptive radiotherapy workflows require images with the quality of computed tomography (CT) for re-calculation and re-optimisation of radiation doses. In this work we aim to improve the quality of on-board cone beam CT (CBCT) images for dose calculation using deep learning. *Approach*. We propose a novel framework for CBCT-to-CT synthesis using cycle-consistent Generative Adversarial Networks (cycleGANs). The framework was tailored for paediatric abdominal patients, a challenging application due to the inter-fractional variability in bowel filling and small patient numbers. We introduced to the networks the concept of global residuals only learning and modified the cycleGAN loss function to explicitly promote structural consistency between source and synthetic images. Finally, to compensate for the anatomical variability and address the difficulties in collecting large datasets in the paediatric population, we applied a smart 2D slice selection based on the common field-of-view (abdomen) to our imaging dataset. This acted as a weakly paired data approach that allowed us to take advantage of scans from patients treated for a variety of malignancies (thoracic-abdominal-pelvic) for training purposes. We first optimised the proposed framework and benchmarked its performance on a development dataset. Later, a comprehensive quantitative evaluation was performed on an unseen dataset, which included calculating global image similarity metrics, segmentation-based measures and proton therapy-specific metrics. *Main results*. We found improved performance for our proposed method, compared to a baseline cycleGAN implementation, on image-similarity metrics such as Mean Absolute Error calculated for a matched virtual CT (55.0 ± 16.6 HU proposed versus 58.9 ± 16.8 HU baseline). There was also a higher level of structural agreement for gastrointestinal gas between source and synthetic images measured using the dice similarity coefficient (0.872 ± 0.053 proposed versus 0.846 ± 0.052 baseline). Differences found in water-equivalent thickness metrics were also smaller for our method (3.3 ± 2.4% proposed versus 3.7 ± 2.8% baseline). *Significance*. Our findings indicate that our innovations to the cycleGAN framework improved the quality and structure consistency of the synthetic CTs generated.

## 1. Introduction

Abdominal irradiation is commonly used in the treatment of young patients with a variety of tumours, including abdominal neuroblastoma and Wilms’ tumour (Bölling *et al* 2010). The success of radiotherapy relies on the accurate delivery of radiation dose to the target volume with minimal toxicity to surrounding normal tissues. Anatomical variations throughout the course of radiotherapy may lead to reduced tumour coverage and increased radiation to healthy tissues, thereby affecting the efficacy of radiotherapy (Berger *et al* 2017). Although approaches to adapt radiotherapy plans have been extensively researched in the head and neck, and pelvis for adult populations (Ghilezan *et al* 2010, Sonke and Belderbos 2010, Thörnqvist *et al* 2013, Morgan and Sher 2020, Tocco *et al* 2020), fewer studies have exclusively focused in abdominal malignancies (Liu *et al* 2012, Schlaich *et al* 2013) especially in younger populations (Laskar *et al* 2015, Guerreiro *et al* 2019). The abdominal and lower abdominal region is particularly susceptible to daily anatomical variations due to the presence of organs with variable filling, including the gastrointestinal (GI) tract, bowel, bladder, and rectum (Berger *et al* 2017). GI air volumes were shown to vary by up to ±80% throughout radiotherapy in adult pancreatic cancer patients (Estabrook *et al* 2018). In children with abdominal cancers, average GI air volume changes of 99.4 ± 126.9 ml (range: 216.7–454.7 ml) have been reported (Guerreiro *et al* 2019) as well as evidence that younger children under anaesthesia are the most predisposed to variability (Lim *et al* 2021, Taylor *et al* 2021). The presence or absence of GI air is reflected in substantial local tissue density changes. Density changes are particularly detrimental to proton beam therapy (PBT) treatments, a favourable radiation modality in abdominal paediatric cancer due to its tissue-sparing capabilities and potential for reducing long term side effects (Guerreiro *et al* 2019, Lim *et al* 2021, Taylor *et al* 2021). A study on adult cervical cancer patients receiving PBT found correlations between dose degradation and volume, thickness, and width of bowel gas (Berger *et al* 2017). The impact of GI air variation on radiotherapy plan robustness was shown to be more pronounced in PBT than x-ray intensity modulated arc therapy plans in both paediatric and adult cancers (Mondlane *et al* 2017, Ashida *et al* 2020, Lim *et al* 2021).

Image-guided radiotherapy (IGRT) technologies, such as cone-beam-CT (CBCT), provide information of the patients’ anatomy immediately before treatment. IGRT enables the monitoring of patient’s anatomical variations between when their planning CT was acquired and subsequent treatment fraction delivery sessions, and potentially allows one to adjust the treatment to the observed anatomical variations (Nazmy *et al* 2012). A limitation to the direct use of CBCT in adaptive pathways is that the imaging quality of CBCT scans is considered significantly inferior to the planning CT scans in terms of contrast-to-noise ratio and prevalence of imaging artefacts such as streaks. In abdominal scans, streak artifacts can be attributed (amongst others) to x-ray scatter and internal motion (Siewerdsen and Jaffray 2001, Peroni *et al* 2012). This has led to growing interest in developing methodology to make the quality of CBCT scans comparable to that of CT.

The most established technique to generate synthetic CTs (synCT) scans with the image quality of CT is based on deformable image registration (DIR) (Giacometti *et al* 2020), in which the planning CT is deformed to match the CBCT’s geometry (Peroni *et al* 2012, Veiga *et al* 2014, Landry *et al* 2015). The main disadvantage of DIR-based approaches is that they cannot properly account for non-deformable changes between consecutive scans, such as collapsing lungs or variable GI air volume and location. Post-processing methods may be paired with DIR to minimize gross anatomical mismatch to a certain degree (Veiga *et al* 2016). Most DIR algorithms also do not meet speed requirements to be usable in real-time applications, particularly without GPU implementations (Shams *et al* 2010, Fu *et al* 2020). An alternative approach is to directly apply scattering-corrections to CBCT images (Mainegra-Hing and Kawrakow 2010, Park *et al* 2015, Hansen *et al* 2018). Furthermore, in recent years deep learning (DL) became an emerging and active field of research for medical image synthesis tasks such as CBCT-to-CT translation (Yu *et al* 2020, Wang *et al* 2021). Compared to classical approaches, data-driven methods have shown encouraging performance metrics and can be applied to unseen datasets quickly. Their main disadvantages are the significant efforts required for appropriate data collection and its curation (Wang *et al* 2021).

There is a wide breadth of previous work on using DL in image-to-image translation. The most popular application of such task is MRI-to-CT conversion (Florkow *et al* 2020, Maspero *et al* 2020a). There are also successful implementations of DL-based solutions for PET attenuation correction (Ladefoged *et al* 2018) and CT synthesis from CBCT in the context of adaptive radiotherapy (Kurz *et al* 2019, Liu *et al* 2020). Different DL frameworks have been proposed for CBCT-to-CT conversion, such as paired UNets (Kida *et al* 2018, Landry *et al* 2019, Li *et al* 2019a, 2019b, Chen *et al* 2020), paired pix2pix generative adversarial networks (GANs) (Zhang *et al* 2021b), paired cycle-consistent GANs (cycleGANs) (Harms *et al* 2019, Eckl *et al* 2020, Liu *et al* 2020, Zhang *et al* 2021b), and unpaired cycleGANs (Kurz *et al* 2019, Liang *et al* 2019, Gao *et al* 2021). While all methods use a data-driven approach to map image intensities between different imaging modalities, unpaired cycleGANs frameworks are of particular interest as they do not require pairs of data with structural correspondence for training but still offer good synthesis performance. This is of great interest in the context of CBCT-to-CT synthesis, where the simultaneous acquisition of scans from both modalities is unfeasible in practice, and using scans acquired closely in time reduces but does not eliminate anatomical mismatch. Previous studies implementing paired approaches used CTs and CBCTs acquired on the same day and applied DIR to compensate for residual anatomical mismatch resulting from differences in the patient’s position in the different scanners and potential internal anatomical changes (Chen *et al* 2020). These datasets still do not represent ideally paired examples and may introduce uncertainties in the training and evaluation of the networks. Additional challenges must also be considered when applying DL methodologies to younger populations. IGRT protocols that include regular CT or CBCT imaging are rarely used in children’s treatments (Hua *et al* 2019) due to concerns with the long term side effects associated with diagnostic radiation doses (Alaei and Spezi 2015). Acquiring CT and CBCT so close in time is not routinely performed in adult populations and even harder to justify in younger patients, limiting the amount of data available for training in a methodology whose performance is well-known to benefit from larger datasets (Shorten and Khoshgoftaar 2019, Brown *et al* 2020).

Promising results were reported by several groups in the application of unpaired cycleGANs for CBCT-to-CT synthesis (Kurz *et al* 2019, Liang *et al* 2019, Maspero *et al* 2020a, Uh *et al* 2021). However, there are still challenges in achieving CT-like quality in synthetic images and completely removing CBCT artefacts. A well-known limitation of unpaired cycleGANs in medical image synthesis is that structural consistency between source and synthetic images cannot be guaranteed, leading to incorrect anatomical information in the synthetic images. Therefore, the original cycleGAN framework is not well suited for CBCT-to-CT synthesis without addressing this limitation.

Data from younger cohorts require techniques specifically developed to account for the variability found in this patient group due to disease, presentation, growth, and development from young age to adulthood. Paediatric patients are a very diverse population, which likely reflects into a more challenging learning task (Ladefoged *et al* 2018, Florkow *et al* 2020, Maspero *et al* 2020b). Childhood cancer is also a rare disease, making it more difficult to gather data from large, representative cohorts across all age groups for DL applications (Guerreiro *et al* 2019). CBCT imaging frequency may vary greatly between different hospitals and types of radiotherapy used, often at the discretion of the treating physician (Nazmy *et al* 2012). Low yearly patient numbers, combined with challenges in collecting imaging datasets in children, make the availability of large datasets scarce, particularly for single institutions (Florkow *et al* 2020). To address limitations in data available for disease specific cohorts, combining multiple datasets from different anatomical sites has been previously proposed; however, this has been achieved simply by including a well-balanced number of cases per patient group in training and evaluation (Maspero *et al* 2020a, Uh *et al* 2021). Transfer learning from adult cohorts is a viable option as well (Ladefoged *et al* 2018). However, there are intrinsic differences in the paediatric cancer population in comparison with adults that will likely affect model generalizability. Differences include treatment strategies, such as the common use of shunts and anaesthesia, and the inherent anatomical differences across developmental stages such as variation in composition and shape of tissues and organs (White *et al* 1991, Bolch *et al* 2020).

Key challenges remain that impede the usability of cycleGANs for CBCT-to-CT synthesis in clinical settings. These challenges include how to ensure the preservation of structural consistency in the synthetic images while removing unwanted artifacts, how to achieve large and representative sample sizes for training — particularly in scarce data settings (such as paediatrics), and how to define adequate ground-truths for the validation of novel synthesis methods when paired data is not available. In this work we propose and evaluate a novel framework for CBCT-to-CT synthesis tailored for paediatric abdominal patients, a challenging application both due to inter-fractional variability in gastrointestinal filling and small patient numbers. This study focuses on exploring improvements to the original cycleGAN framework and training data selection techniques aiming to addresses the outlined challenges in the proposed application. Preliminary results of this study were presented in conference publications (Szmul *et al* 2021a, Szmul *et al* 2022). The key novel aspects of our framework are: (1) application of a global residuals only learning approach, (2) incorporating structural consistency metrics to promote anatomical plausibility of synthesized images, (3) a novel smart data selection process to efficiently combine data from multiple patient groups (weakly paired approach), and (4) an automated pipeline for the quantitative evaluation of synthetic images.

## 2. Methods and materials

### 2.1. A framework for CBCT-to-CT synthesis using cycleGANs

**Figure 1.**
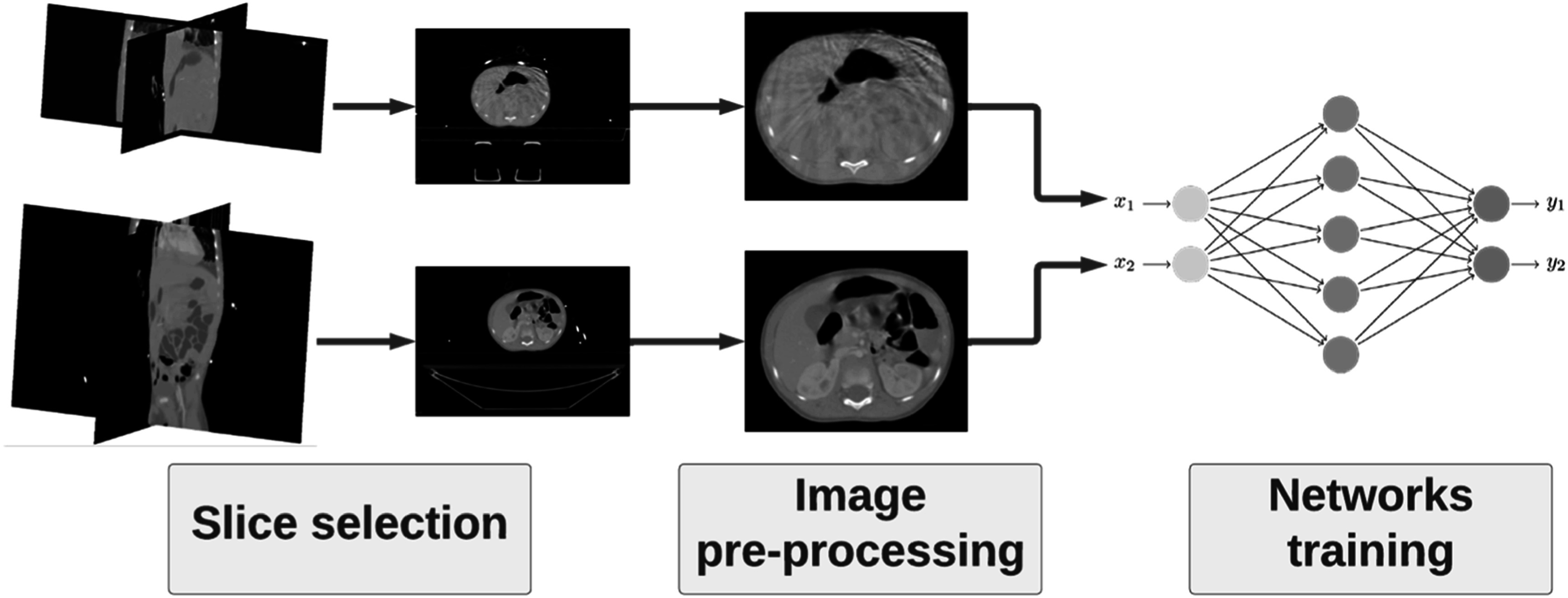
Overview of the CBCT-to-CT synthesis framework developed, highlighting the three main steps: training data selection, image pre-processing and training of the networks.

The synthetic CT (synCT) generation pipeline developed consisted of the following key steps: (1) smart slice selection strategy, (2) image pre-processing and (3) network training and inference (figure 1).

#### 2.1.1. Smart slice selection via weakly paired data approach

**Figure 2.**
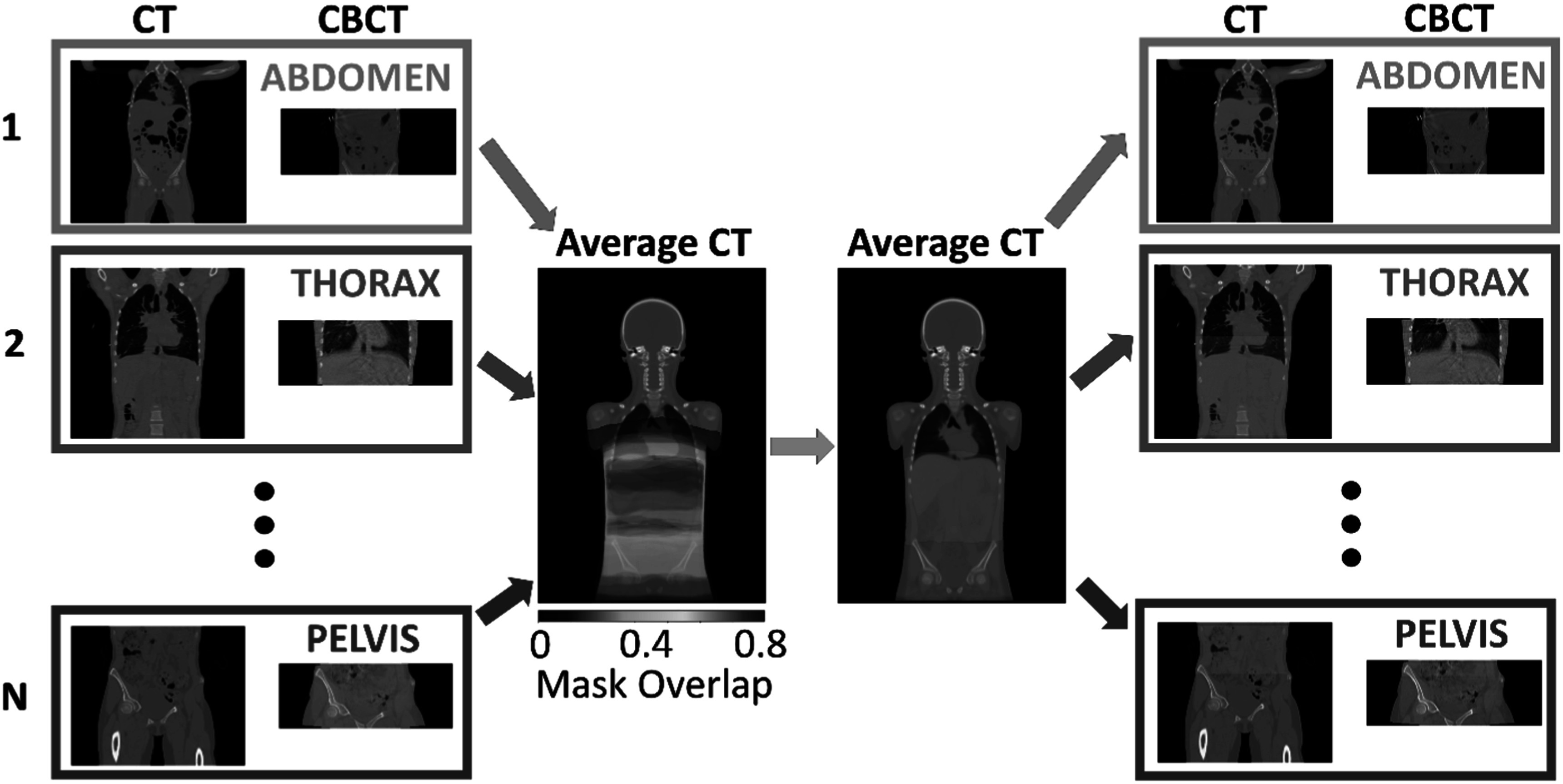
Overview of the proposed weakly paired data approach. The CT and CBCT scans were spatially normalized to a paediatric anatomical atlas, and a common field-of-view mask generated after fusing the CBCT body contours. The mask was then mapped back to each individual space and used to sample for training only CT and CBCT slices within the region of interest. This approach effectively adjusted the field-of-view of all scans to the abdominal region only, allowing us to best utilize data from multiple patient groups.

CT and CBCT scans do not cover the same sections of the body due to the reduced field-of-view of CBCT (in comparison to CT) and the intra-patient variability in the location of the imaging isocentre. We propose a weakly paired data approach to compensate for the intra and inter-subject variability in imaged anatomical location. The CT and CBCT scans were spatially normalized to a common reference space and only slices from the same body regions were sampled (figure 2). We used as reference space an atlas-based paediatric average anatomy and a co-registration strategy developed and evaluated in our previous study (Veiga *et al* 2021). A region of interest was defined by fusing the co-registered CBCT body contours on the average space and applying a thresholded majority voting. The created common field-of-view mask was then propagated back to each subject’s space. Slices in the individual CT/CBCT scans located outside of this mask were excluded from all experiments. The top and bottom four slices of each CBCT scans were typically truncated and thus were also excluded from all experiments.

#### 2.1.2. Image pre-processing steps

Pre-processing steps were applied to all CT and CBCT scans before presenting them to the networks for training and inference. The images were corrected to exclude surrounding equipment and elements external to the subject —such as the treatment couch, anaesthesia equipment, shunts, feeding tubes, and/or lines, some of which may introduce high-intensity artifacts. External components were defined as regions outside the body contour and were replaced with air equivalent intensity (−1000 HU). Internal regions with high-intensity artefacts were segmented by applying thresholding (HU $\geqslant \,$1700) followed by morphological operations, and subsequently overwritten with water equivalent intensities (0 HU). Finally, the image intensities were clipped to the range of [−1000, 1000] and normalized to [−1, 1]. The proposed adjustments of intensities aimed at preventing the networks from generating elements such as tubes and internal lines in the synthetic images that were not present in the source images but commonly present in the training data.

To account for significant variation in body size and shape across ages, an axial normalisation pre-processing step was also employed. The training CT/CBCT slices were axially normalized by fitting the body contour to a fixed size of 256 × 256 and resizing the corresponding image slice. This step was done by finding the longest profile between *x* and *y* axis on each slice; a margin of 10 pixels padded with −1000 HU was included to each slice to allow for additional variation in shape during data augmentation. The determined distance was used to calculate the required scaling factor to best fit the slice to the fixed image size of 256 × 256. The same scaling factor was applied on both directions to preserve body shape. The images requiring resampling were interpolated using spline interpolation followed by intensity clipping to ensure the intensity ranges do not extend the normalisation ranges. The spatial normalisation of the body aimed at artificially reducing the anatomical variability in size across the population while preserving shape variability. In our preliminary investigations we have found consistent improvements by including axial normalisation — without this step the resulting synCTs were often unrealistic and the body contours could be distorted, particularly for smaller patients (Szmul *et al* 2021a). Our observations were also confirmed by Uh *et al* (2021).

#### 2.1.3. Design of the cycleGAN network

**Figure 3.**
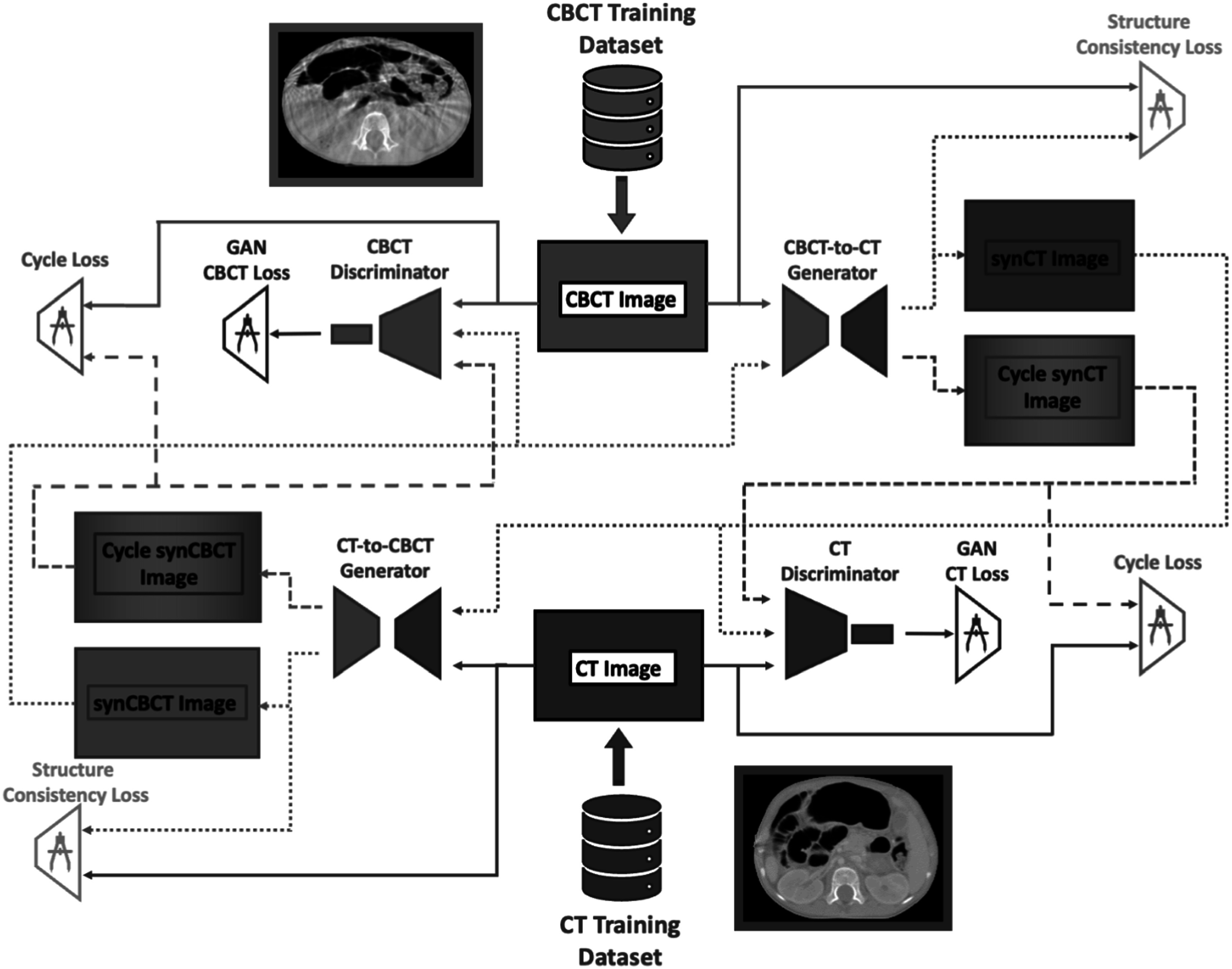
Overview of the proposed cycleGAN architecture. The main difference from the original cycleGAN implementation was the inclusion of the Structure Consistency Loss between raw input images and their synthetic contour parts, shown in orange colour. The paths of the different types of images are shown with dedicated line types: solid for the raw images, dotted for synthetic images and dashed for cycle images. Blue and red colours correspond to the modality arm paths, blue to the CBCT arm path and red to the CT arm path. For clarity the identity loss was omitted from the diagram.

A 2D cycleGAN approach for CBCT-to-CT synthesis was implemented in this study, known for its good performance in unpaired data style conversion (figure 3). We followed closely the implementation presented in Zhu *et al* (2017). The cycleGAN framework consists of two arms with a pair of a generator and a discriminator in each of the arms. One arm converts CBCT to CT, and the counterpart generates CBCT from CT. The task of a generator in this configuration is to, conditioned on an input image from one modality, provide a corresponding image in another imaging modality.

We investigated two different generator architectures: UNet-based (Ronneberger *et al* 2015) and ResNet-based (He *et al* 2015). The ResNet-based generator is, similarly to UNet, an encoder-decoder architecture. However, it does not apply skip connections and uses residual blocks at the bottleneck stage. The implementation was based on works of Johnson *et al* (2016) and Isola *et al* (2018), and has been successfully used in applications such as style and domain adaptation. For the rest of the paper, we will refer to these as UNet and ResNet generators respectively, to keep it consistent with the cycleGAN implementation of Zhu *et al* (2017). The choice of widely used generator architectures allowed our study to focus on improvements to the synthesis framework only.

**Figure 4.**
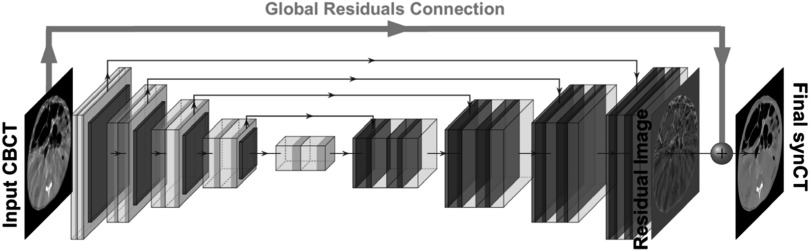
Visualization of the concept of the global residuals only learning, by applying the global residual connection which combines the input image with the output of the generator. For illustrative purposes the global residuals only were shown for the UNet architecture, but the same concept was applied to the ResNet architecture.

A novel aspect of our cycleGAN CBCT-to-CT synthesis framework was incorporating the concept of global residuals only learning in the generators. Global residual only learning is a common technique in computer vision image restoration (Zhang *et al* 2021a), denoising (Zhang *et al* 2017) and enhancement (Kim *et al* 2016). Unlike other pairs of very distinct imaging modalities (for example, CT and MR), CBCT and CT share many acquisition similarities such that CBCT may be considered as a distorted CT image to a certain extent. Therefore, instead of learning the whole image, our proposed network focused only on predicting the unwanted elements in the images, which were then combined with the source image to produce the synthetic counterpart (figure 4). This approach effectively redefines the network’s aim from synthesizing to refining/enhancing images. The proposed concept of global residuals was applied to both generator architectures.

The task of the discriminators is to distinguish between real and synthetic images implemented as binary classifiers with a binary cross-entropy loss function. The core loss in the framework is a GAN loss function (${ {\mathcal L} }_{\mathrm{GAN}}$), which is used to reward the generator for delivering outputs closer to the target domain, while the discriminator is rewarded for distinguishing between real and generated data. We applied an extended version of the loss using Least Squares GAN loss functions (${ {\mathcal L} }_{\mathrm{GAN}}$) which also notes how far from the decision boundary the new generated image was, when evaluated by the discriminator (Mao *et al* 2017). CycleGANs utilise two arms, synthesizing CT into CBCTs and CBCT into CT, therefore there were two losses corresponding to each arm. The cycle consistency loss (${ {\mathcal L} }_{\mathrm{cycle}}$) evaluates how similar the input image is to itself after going through both generators. This loss indirectly promotes structural consistency between source and synthetic images but cannot guarantee it (Ge *et al* 2019a, 2019b). Consequently, studies have reported for example differences in body outline in CT images generated with CBCT-to-CT cycleGAN methodology (Kurz *et al* 2019). To promote preservation of the CBCT anatomy, the original formulation was here extended by introducing a structure consistency loss (${ {\mathcal L} }_{\mathrm{structure}}$) in the form of locally normalized cross correlation (LNCC) (Hermosillo *et al* 2002) between the original images and their synthesized counterparts. A similar approach was applied by Hiasa *et al* (2018a) in multimodal image synthesis, where cross correlation was calculated between gradients of CT and MRI images. In our case, before the LNCC was calculated, the images were smoothed with gaussian filter and gaussian noise was added to focus the attention of the measure into higher level structures. Additional regularisation was promoted by the identity loss (${ {\mathcal L} }_{\mathrm{identity}}$), which penalises applying changes to an image introduced to the generator if the image is already in the target domain (for instance, the output of the CT-to-CBCT generator when a CBCT image is the input should be the same image). L1 was used as the identity and cycle consistency losses. The overall proposed cycleGAN framework was optimised based on the total loss function stated in equation (1). Complete details of each loss function can be found in supplementary data A.

\begin{eqnarray*}\begin{array}{c} {\mathcal L} \left({G}_{\mathrm{CT}\to \mathrm{CBCT}},{G}_{\mathrm{CBCT}\to \mathrm{CT}},{D}_{\mathrm{CT}},{D}_{\mathrm{CBCT}}\right)\,=\\ { {\mathcal L} }_{GA{N}_{CT\to CBCT}}\left({G}_{\mathrm{CT}\to \mathrm{CBCT}},{D}_{\mathrm{CBCT}}\right)\\ \,+\,{ {\mathcal L} }_{GA{N}_{CBCT\to CT}}\left({G}_{\mathrm{CBCT}\to \mathrm{CT}},{D}_{\mathrm{CT}}\right)\\ \,+\,{\lambda }_{\mathrm{cycle}}{ {\mathcal L} }_{\mathrm{cycle}}\left({G}_{\mathrm{CBCT}\to \mathrm{CT}},{G}_{\mathrm{CT}\to \mathrm{CBCT}}\right)\\ \,+\,{\lambda }_{\mathrm{identity}}{ {\mathcal L} }_{\mathrm{identity}}\left({G}_{\mathrm{CBCT}\to \mathrm{CT}},{G}_{\mathrm{CT}\to \mathrm{CBCT}}\right)\\ \,+\,{\lambda }_{\mathrm{structure}}{ {\mathcal L} }_{\mathrm{structure}}\left({G}_{\mathrm{CBCT}\to \mathrm{CT}},{G}_{\mathrm{CT}\to \mathrm{CBCT}}\right).\end{array}\end{eqnarray*}

### 2.2. Data and data split

Scans from 63 patients aged 2 to 24 years old historically treated with radiation therapy were used in this study. The data for this study was requested and approved in line with the internal information governance procedures of the University College London Hospital NHS Foundation Trust Radiotherapy Department. The smart slice selection process described in section 2.1.1 allowed us to make use of data from multiple treatment groups. Thus, we included not only subjects irradiated to the abdomen (68%) but also to the thoracic (8%) and pelvic (24%) regions to increase the dataset size for training in this scarce data domain. One planning CT and one to ten weekly CBCTs were gathered per patient, from a variety of scanners and on-board imaging systems. The planning CTs were acquired on a SOMATON Confidence (Siemens), LightSpeed RT16 or Discovery 710 (GE Medical Systems) with 120 kVp and field of view of 28 – 50 cm, resulting in reconstructed images with 0.93 (±0.1) × 0.93 (±0.1) × 2.29 (±0.25) mm^3^ resolution. Contrast enhancement was used in 70% of the CT scans. The CBCTs were acquired with the on-board imaging of the Varian Medical Systems Truebeam or Clinac with 125 kVp, 15 – 80 mA, 13 – 18 ms, half-fan mode, and shifted panels. The CBCT scans had a field of view of 41 – 46 cm and were reconstructed with a resolution of 0.91 (±0.1) × 0.91 (±0.1) × 1.99 (±0.001) mm^3^. In total 63 CT and 209 CBCT scans were available for the development and evaluation of the proposed CBCT-to-CT synthesis framework. In addition to the CT and CBCT scans, corresponding segmentations of the body, GI air, bone, and lung volumes were used for evaluation purposes. The volumes were first semi-automatically generated, and then manually edited and revised slice-by-slice using ITK-Snap (Yushkevich *et al* 2006). Post-processing was applied to all contours to reduce common manual segmentation errors, such as discarding small volumes located outside the body.

The whole dataset was divided into 50 development cases and 13 cases for testing. The development dataset contained scans from thoracic-abdominal-pelvic subjects and was randomly split as 40 and 10 for training and validation. The testing dataset consisted of scans from abdominal subjects only, and cases were selected to achieve a well-balanced representation of ages and genders, while prioritizing the use of cases with multiple CBCTs for development purposes. The split between the datasets was made based on non-image characteristics only to minimise selection bias (i.e. the scans were not visually inspected during the splitting process).

We have created three training datasets by applying different slice selection approaches to our imaging data, which resulted in different number of slices being used for training. In the first dataset no data selection threshold was applied (naïve sampling). Two other datasets were created using the smart data selection via weakly paired approach, with two different thresholds to create the datasets: 1% and 40%. The 1% mask excluded rarely represented slices (hereby referred as ‘no outliers’) in a systematic way (such as slices containing the neck or inferior members), which could accidently be included in the naïve sampling. The 40% mask effectively adjusted the field-of-view of all images to the abdominal region, as shown previously in figure 2 (hereby referred as ‘smart data selection’). A complete breakdown of the number of CT/CBCT slices included per approach can be found in supplementary data B.

### 2.3. Network training and inference

We trained a total of 16 cycleGAN frameworks by varying the generator architectures (UNet and ResNet), the slice selection strategy (naïve, no outliers and smart data selection), with and without global residuals, and including (or not) the structure consistency loss in the frameworks.

For the ResNet networks we followed the implementation used in Johnson *et al* (2016) and Isola *et al* (2018). It consisted of a down-sampling (encoder-like) section, 9 residual blocks at the bottleneck stage, and an up-sampling (decoder-like) section afterwards. The UNet-like architecture was a standard implementation by Ronneberger *et al* (2015), with 5 down/up sampling levels, Leaky ReLu activation function used in the downspampling blocks and ReLu in upsampling blocks, following Isola *et al* (2018). Both architectures were used with 64 initial filters, instance normalisation and dropout probability set to 0.5. The implementation of the discriminator followed PatchGAN discriminator with 70 × 70 overlapping patches, initially introduced in Isola *et al* (2018) and successfully applied by Uh *et al* (2021). The discriminator had 3 layers of depth, with 64 filters in the initial layer. Leaky ReLu was applied as the activation function. The cycleGAN frameworks were trained for 200 epochs with a batch size of 4 and diminishing learning rate on an in-house high-performance computing (HPC) facility with graphical processor units (Titan X 12GB GPU cards). The initial learning rate was set to 0.002 for the initial 100 epochs and was linearly decreased to 0 for the following 100 epochs. Adam optimiser was used with momentum *β*_1_ = 0.5. Before calculating the Structure Consistency Loss, the images were smoothed with gaussian filter (kernel size 7, *σ* =1) and random gaussian noise was added (with the magnitude of 0.001). The losses were calculated with fixed weights for all experiments: *λ*_cycle_ = 10, *λ*_identity_ = 0.5, *λ*_structure_ = 1 (when no structure consistency was used this weight was set to 0). For data augmentation, the following transformations were applied: random flipping (left and right), elastic deformations with the spacing set to (64, 64) and magnitude range to (3, 3), rotations by up to 15 degrees, and gamma contrast adjustments in a range [0.7, 1.3]. The probability of all random augmentations was set to 0.5.

At the inference stage, unseen datasets were fed to the network subjected to the same pre-processing steps as the training data (section 2.1.2). The generated synCT were then resized back to their original size, their intensities were rescaled to [−1000, 1000] and stacked back into a 3D volume for evaluation.

### 2.4. Evaluation experiments

#### 2.4.1. Definition of the ground-truth

**Figure 5.**
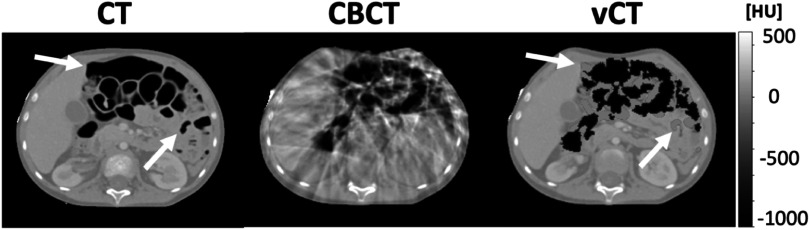
Example of paired CT, CBCT and virtual CT slices. The virtual CT was created by registering CT to CBCT and semi-automatically correcting for GI air differences. The air regions inside body in CT were replaced with water equivalent values prior to the registration, while air regions from CBCT were subsequently embedded into the virtual CT (that is indicated by white arrows in CT and in corresponding places in vCT).

Evaluating the quality of synthetic images is a challenging task due to the lack of a real ground-truth, which in this application would consist of pairs of simultaneously acquired CT and CBCT scans. Thus, we opted to evaluate the synCTs against two complementary image ground-truths: the raw CBCT and a virtual CT (vCT) matched to the anatomy of the raw CBCT. The vCT consisted of the planning CT deformably registered to the CBCTs using the open-source NiftyReg (Rueckert *et al* 1999, Ourselin *et al* 2001, Modat *et al* 2010, 2014) with additional post-processing steps to account for the variable position of the GI air between scans (complete details in supplementary data C). Figure 5 shows an example of a planning CT and corresponding CBCT and vCT. The use of those two ground-truths enabled the quantitative evaluation of both anatomical and intensity consistency of the synthetic images.

#### 2.4.2. Experiments

A total of three sets of experiments were conducted to optimise and evaluate the proposed CBCT-to-CT synthesis method.

*Experiment 1: Ablation study of the proposed modifications.* We performed a general search for the optimal configuration of the framework on one-fold of the development dataset to find the optimal CBCT-to-CT synthesis framework. A total of 16 framework configurations were quantitatively evaluated calculating global image similarity metrics between the gold-standards and the synthesized images (details in section 2.4.3).

*Experiment 2: Five-fold cross validation and benchmarking of the optimal configuration.* We evaluated in more detail the optimal configuration of the framework on five-folds of the development dataset and compared it against a baseline configuration. These experiments aimed to ascertain on the level of overfitting of the method to the training dataset. The proposed cycleGANs was benchmarked against a baseline implementation of the cycleGANs (also defined in Experiment 1). Both methods were evaluated in terms of global image similarity with CBCT and vCT (details in section 2.4.3).

*Experiment 3: Comprehensive quantitative evaluation on the unseen testing dataset.* The baseline and the identified optimal configuration were retrained on the whole development dataset and used for inference in the testing dataset. This experiment aimed to evaluate how the model generalises to unseen data, and to validate its performance in detail in the proposed application. The proposed cycleGAN was again compared to a baseline configuration of the cycleGAN. The performance of the networks was assessed through global image similarity metrics, segmentation-based measures and radiotherapy-specific metrics (details in section 2.4.3).

#### 2.4.3. Evaluation metrics

The quality of the CBCT-to-CT synthesis achieved by different methods was evaluated using metrics that quantify how close the synthetic CT is to the original planning CT intensities and to the CBCT anatomy. These metrics may be grouped into three separate categories: (1) global image similarity (experiments 1–3), (2) segmentation-based measures (experiment 3) and (3) radiotherapy-specific metrics (experiment 3). Global image similarity metrics as well radiotherapy-specific metrics were calculated in 2D (i.e. slice by slice) in all experiments, while segmentation-based metrics were calculated in 3D. The metrics are briefly described in the following paragraphs.

We used three global image similarity metrics when comparing pairs of images: mean absolute error (MAE), normalised cross-correlation (NCC) and root mean square error (RMSE). Those measures were calculated between the evaluated image and two ground-truth images: CBCT and vCT. The metrics were chosen to provide complementary information on the similarity between synCTs in pixel intensities (MAE and RMSE) and structural agreement (NCC) with the CBCT/vCT. It is expected that MAE and RSME between synCT and CBCT will always reflect some disagreement from locally incorrect CBCT intensities. However, they may also reflect differences due to structural inconsistencies. Therefore, due to their complementary nature, all metrics were reported and analyzed for both ground-truths. Voxels outside the body contour were excluded from analysis; the intensities of all images were clipped to [−1000, 1000].

For segmentation-based evaluation, we used complementary measures of volume similarity between different types of tissue within the body contour: GI air, bones, soft tissues (muscles and fat) and lungs. Ground-truth segmentations of these volumes on CBCTs were compared with corresponding structures automatically segmented on the synthetic CTs using the Dice similarity coefficient (DSC), the Hausdorff distance (HD) and average pixel intensity (HU) as metrics. To automate the tissue segmentation we implemented and trained a patch-based 3D-UNet (Çiçek *et al* 2016) using the MONAI library (https://monai.io/) (Consortium 2020) (complete implementation details in supplementary data D). The training dataset consisted of a total of 183 CBCT and 50 CT scans, and corresponding ground-truth labels, from the same subjects included in development dataset of the cycleGAN networks. The training dataset consisted of both CT and CBCT images. The assumption was that while synCTs were expected to have CT-like quality, some features/artifacts typical of CBCT may not be completely removed. Our initial experiments using only CTs led, for example, to gross segmentations errors in the presence of streak artifacts caused by high-intensity elements.

For radiotherapy-specific evaluation, we calculated polar water equivalent thickness (WET) differences between the vCT and synthetic CT methods (${\mathrm{\Delta }}{\mathrm{WET}}$). The WET is the thickness of water that would cause a proton beam to lose the same energy as if it had crossed a certain medium. The WET for a given beam direction (${\mathrm{\Theta }}$) was calculated as:

\begin{eqnarray*}{\mathrm{WET}}^{{\mathrm{\Theta }}}=\displaystyle \sum _{i,j,k\in S}{\mathrm{RSP}}_{i,j,k}\times {d}_{i,j,k},\end{eqnarray*}

where $S$ is a set of voxels that contains the beam path, ${\mathrm{RSP}}_{i,j,k}$ is the relative stopping power (estimated from CT numbers using a standard calibration curve), and ${d}_{i,j,k}$ is the path length of the beam inside voxel $\left(i,j,k\right)$ estimated by a ray tracing algorithm (Zhang *et al* 2010, Lui 2018). The WET between the beam entrance point and centre-of-mass of the body contour was calculated slice-by-slice, considering a complete arc with steps of one degree. The WET differences (${\mathrm{\Delta WET}}_{A,B}^{{\mathrm{\Theta }}}$) between two scans (A and B, where A is the ground-truth) was reported as the RSME value:

\begin{eqnarray*}{\mathrm{\Delta WET}}_{A,B}^{{\mathrm{\Theta }}}=\mathrm{RMSE}\left\{100\times \frac{{\mathrm{WET}}_{B}^{{\mathrm{\Theta }}}-{\mathrm{WET}}_{A}^{{\mathrm{\Theta }}}}{{\mathrm{WET}}_{A}^{{\mathrm{\Theta }}}}\right\}.\end{eqnarray*}

$\mathrm{\Delta WET}\,$was calculated overall and for each gantry angle individually, to quantify the impact that synthesis errors have on calculation of clinical dose distributions and ascertain if some beam angles were more affected than others.

## 3. Results

### 3.1. Experiment 1: Ablation study of the proposed modifications

Table 1 presents the numerical results in terms of the global similarity measures for the ablation study of the proposed modifications, where we tested a total of 16 configurations for the network. We systematically added the proposed modifications (global residuals learning, smart data selection and structure consistency loss) and observed steadily improved performance of the framework, regardless of the generator architecture that was used. The largest improvements were observed by introducing the global residuals to the generators. Configurations that used the UNet architecture always outperformed the ResNet-based frameworks.

**Table 1.** Results of the ablation study of the proposed modifications in terms of global similarity metrics for two generator networks architectures. The proposed and baseline configuration are highlighted in bold font.

Architecture	Global residuals	Data Selection	Structure consistency	MAE_vCT [HU]	NCC_vCT [1]	RMSE_vCT [HU]	MAE_CBCT [HU]	NCC_CBCT [1]	RMSE_CBCT [HU]
ResNet	No	None	No	78.9 ± 30.0	0.91 ± 0.06	148.0 ± 62.4	86.8 ± 31.5	0.91 ± 0.06	148.1 ± 60.8
ResNet	No	No outliers	No	71.7 ± 21.7	0.92 ± 0.04	136.7 ± 38.3	71.8 ± 18.6	0.92 ± 0.04	130.2 ± 34.2
ResNet	Yes	None	No	58.7 ± 15.8	0.96 ± 0.02	102.3 ± 29.8	63.8 ± 17.3	0.96 ± 0.02	107.3 ± 31.3
ResNet	Yes	None	Yes	54.5 ± 15.5	0.97 ± 0.01	90.5 ± 22.4	48.5 ± 11.9	0.97 ± 0.02	87.8 ± 25.9
ResNet	Yes	No outliers	No	61.9 ± 17.5	0.9 ± 0.05	98.3 ± 25.3	71.0 ± 16.4	0.92 ± 0.04	95.0 ± 20.8
ResNet	Yes	No outliers	Yes	54.2 ± 17.0	0.92 ± 0.04	84.3 ± 23.6	40.2 ± 10.3	0.96 ± 0.02	58.7 ± 14.5
ResNet	Yes	Smart data selection	No	59.4 ± 13.9	0.96 ± 0.01	101.9 ± 21.3	69.2 ± 13.9	0.95 ± 0.02	107.9 ± 23.3
ResNet	Yes	Smart data selection	Yes	53.5 ± 16.0	0.97 ± 0.01	90.7 ± 22.2	51.9 ± 12.6	0.97 ± 0.02	90.8 ± 24.9
UNet	No	None	No	61.2 ± 23.3	0.95 ± 0.05	110.6 ± 48.7	63.9 ± 26.4	0.94 ± 0.05	111.7 ± 50.7
**UNet**	**No**	**No outliers**	**No**	**59.2 ± 15.2**	**0.95 ± 0.03**	**107.1 ± 29.1**	**59.6 ± 18.9**	**0.95 ± 0.03**	**104.9 ± 34.2**
UNet	Yes	None	No	54.4 ± 12.8	0.97 ± 0.01	90.0 ± 20.0	61.4 ± 16.3	0.96 ± 0.02	97.1 ± 28.0
UNet	Yes	None	Yes	53.1 ± 15.3	0.97 ± 0.01	87.5 ± 21.9	47.3 ± 14.1	0.97 ± 0.02	85.8 ± 28.4
UNet	Yes	No outliers	No	54.1 ± 14.1	0.97 ± 0.02	89.7 ± 22.9	56.9 ± 16.9	0.96 ± 0.02	93.6 ± 31.3
UNet	Yes	No outliers	Yes	53.0 ± 15.1	0.97 ± 0.01	87.6 ± 21.8	47.0 ± 14.8	0.97 ± 0.02	85.3 ± 28.6
UNet	Yes	Smart data selection	No	52.3 ± 12.4	0.97 ± 0.01	86.4 ± 19.6	57.0 ± 16.3	0.97 ± 0.02	92.2 ± 29.4
**UNet**	**Yes**	**Smart data selection**	**Yes**	**51.7 ± 15.0**	**0.97 ± 0.01**	**85.0 ± 20.9**	**45.0 ± 12.6**	**0.97 ± 0.02**	**82.8 ± 27.5**

MAE vCT/CBCT — Mean absolute error calculated with respect to vCT/CBCT; NCC vCT/CBCT — Normalised cross correlation calculated with respect to vCT/CBCT; RMSE vCT/CBCT — Root mean square error calculated with respect to vCT/CBCT.

This first experiment allowed us to narrow down into an optimal configuration, as well as a baseline configuration for comparison purposes, to use in the following experiments. The optimal configuration used the following settings: UNet generator with structure consistency loss and global residuals learning trained with the ‘smart data selection’ strategy. We will refer to this configuration as the ‘proposed’ approach for the rest of the manuscript. Likewise, the ‘baseline’ cycleGAN configuration settings chosen were: UNet generator, without structure consistency loss and global residuals learning trained with the ‘no outliers’ slice selection method.

### 3.2. Experiment 2: Five-fold cross validation and benchmarking of the optimal configuration

**Figure 6.**
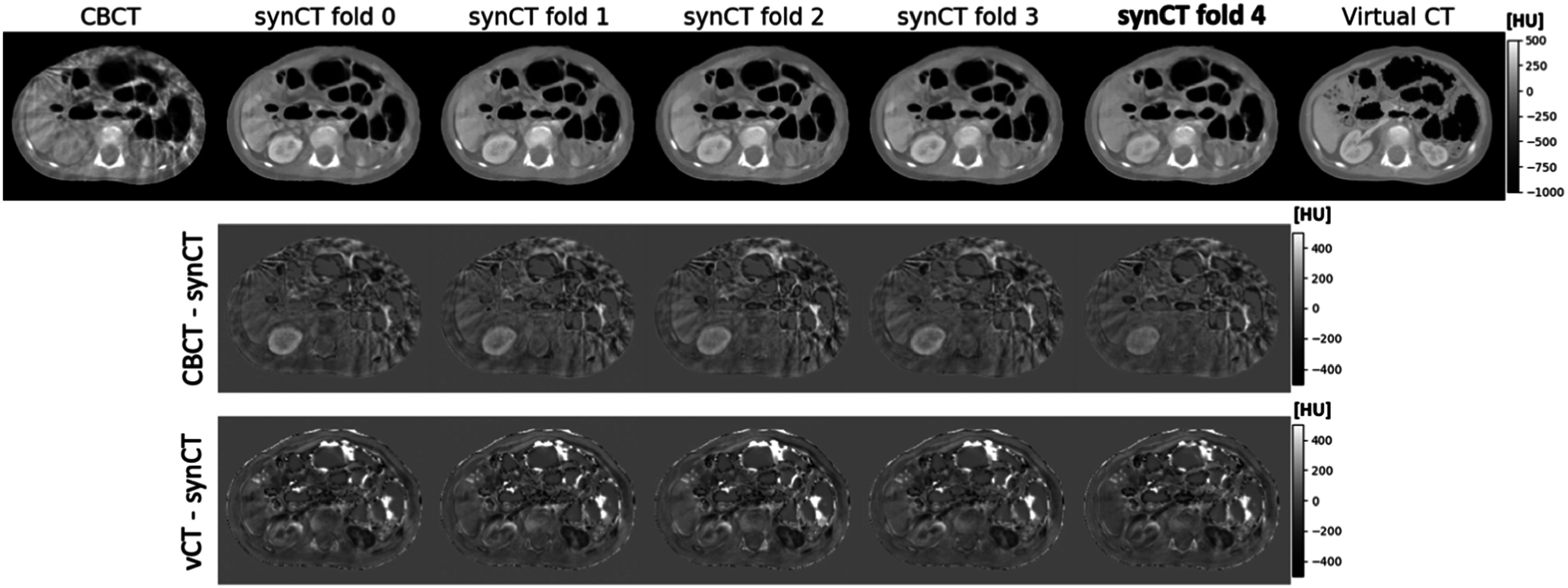
Comparison of synthetic CTs between different folds when 5-fold cross validation was applied. The fold where the slice was part of the validation subset is shown in bold (fold 4). For the remaining folds the slice was used for training. No significant visual differences in anatomy were observed. The two lower rows show the difference images between the output synthetic CTsand the CBCT (top) and virtual CT (bottom).

**Figure 7.**
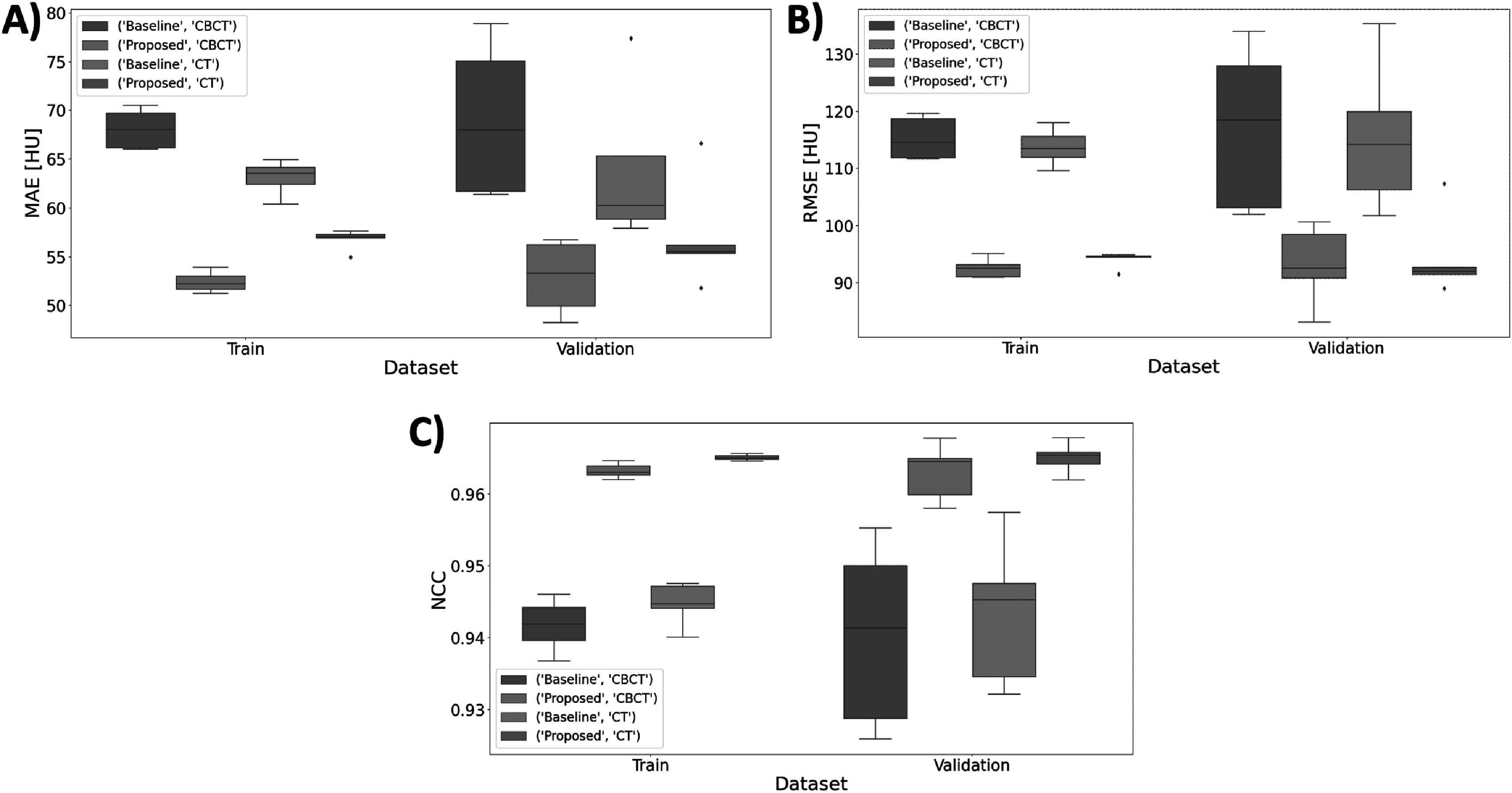
Box plots showing the distribution of the numerical results between the proposed and baseline synCT for mean absolute error (MAE) (A), root mean square error (RMSE) (B) and normalised cross-correlation (NCC) (C) calculated between the synCTs and ground-truths (CBCT and virtual CT).

In the 5-fold cross validation study we investigated the proposed synCT generation configuration against the baseline cycleGAN configuration. The proposed method performed consistently between different folds. Figure 6 shows an example of inference results on the same slice for different folds, where in 4 folds the slice was used for training and in one it was part of the validation subset (4th fold). Only small levels of inconsistency were observed between folds, mostly originating from differences in where contrast enhancement is added by the networks (e.g: brightness of the kidneys and liver). These visual findings were also confirmed with the global similarity metrics, which were consistent for different folds and outperformed considerably the baseline method (figure 7). To ascertain on the level of overfitting of the method to training dataset, the evaluation data was shown for both training and validation datasets — the results between the two datasets were comparable on average, with the validation set having larger standard deviations for all metrics. The proposed method resulted not only in better mean values compared to the baseline method, but also in lower standard deviations. Overall, the numerical data suggested an improved consistency with our method, in agreement with the visual inspection findings.

### 3.3. Experiment 3: Comprehensive quantitative evaluation on the unseen testing dataset

#### 3.3.1. Global image similarity

**Figure 8.**
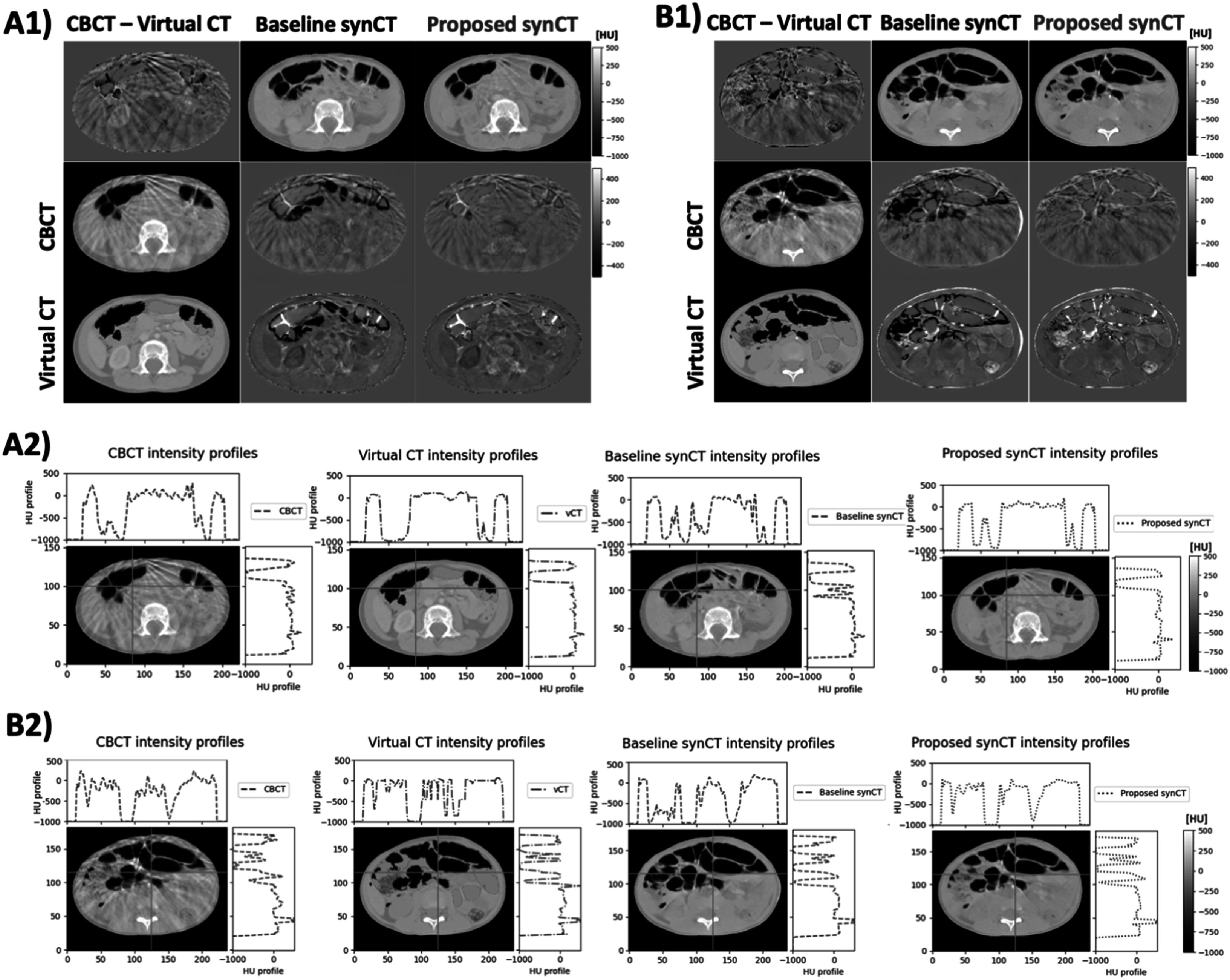
Visual comparison of the results between the proposed and baseline synCTs with respect to the virtual CT and CBCT for two example, cases A and B. The difference images highlight regions of anatomical and intensity disagreement (A1 and B1). The difference between the virtual CT and CBCT highlights the challenge in establishing reliable ground-truth images. For both cases, intensity profiles for cross-sections for the CBCT, virtual CT, baseline synCT and the proposed synCT are also shown (A2 and B2).

The final experiments were conducted after re-training the proposed and baseline configuration on the whole development dataset and applying them to a previously unseen testing dataset. For clarity, due to the smart slice selection step being included, the final proposed network was effectively trained only on slices coming from the abdominal region (although some slices were from patients treated for diseases in different anatomical sites). Figure 8 shows a visual comparison between the two configurations for two example slices from different subjects (A and B). The baseline configuration had worse performance in terms of anatomical realism and consistency when compared to both ground-truths (CBCT and virtual CT). Our proposed method generated synthetic images that more closely matched the anatomy of the source CBCT. Upon visual inspection, the baseline method was found to commonly remove or add vertebrae and/or introducing inexistent bowel pockets, as well as contrast from shunts to the synCTs. Such patterns of failure were not observed for the proposed synCT method, which successfully preserved the anatomy from CBCT while improving the overall image quality. A limitation seen in both synCT methods was that occasionally the generated synCTs were contrast-enhanced, when the original CBCT or matching CT was not (and vice-versa), likely because the training set included scans both with and without contrast agent injection. That has led to inconsistencies in contrast between adjacent slices in some cases. The additional analysis of cross-sectional intensity profiles highlighted how the baseline cycleGAN method was unable to preserve the structural information, adding up inexistent air pockets. The proposed method follows more closely the profiles of the CBCT and virtual CT.

Visual inspection was followed by numerical evaluation in terms of global image similarity metrics. The proposed method achieved better numerical results than the baseline method for all scores (table 2). These results were only slightly worse than those reported for the 5-fold cross validation, indicating that the method did not overfit to the training dataset and generalized well to unseen cases.

**Table 2.** Numerical evaluation of the baseline and proposed method in terms of global image similarity metrics.

	Virtual CT			CBCT		
	MAE [HU]	NCC [1]	RMSE [HU]	MAE [HU]	NCC [1]	RMSE [HU]
Baseline synCT	58.9 ± 16.8	0.96 ± 0.02	102.6 ± 31.7	63.4 ± 15.9	0.95 ± 0.02	110.0 ± 32.2
Proposed synCT	55.0 ± 16.6	0.97 ± 0.02	89.8 ± 23.8	49.8 ± 10.9	0.97 ± 0.02	88.6 ± 24.9

#### 3.3.2. Segmentation-based measures

**Figure 9.**
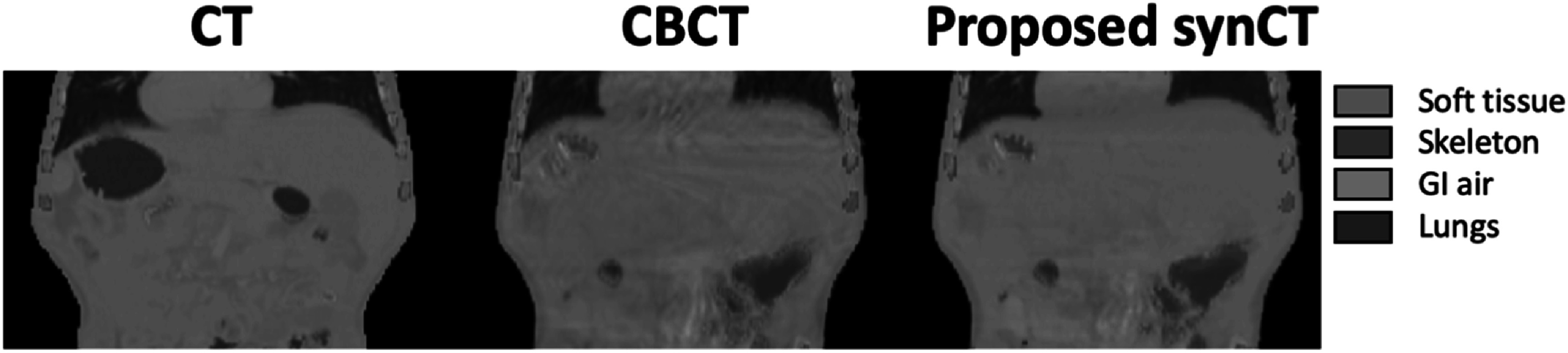
Segmentation results for CT, CBCT, and proposed synCT.

A 3D-UNet segmentation was trained to identify four different types of tissue in CT and CBCT within the body contour: soft tissues, skeleton, GI air and lungs This network was used to automatically segment each testing CT, CBCT and synCT (figure 9).

Table 3 presents the similarity between automated and ground-truth segmentations in terms of DSC, HD and average intensity for each tissue type. The DSC and HD values reported for planning CT and CBCT represent how well the automated segmentation method performed on real images from the testing dataset, with DSC ranging from 0.889 to 0.978 for both scans and tissue types. The DSC and HD values reported for the synCTs allowed to assess their structural similarity to the source CBCTs. The values of DSC and HD reported for the synCT, while expectedly inferior to those on real CT and CBCT, were of similar magnitude indicating that the automated segmentation worked well on all datasets. The proposed synCT approach outperformed the baseline synCT demonstrating better structural similarity to the CBCT segmentations. The average intensity data allowed us to assess the intensity similarity between the synCTs and planning CTs for different tissue types. The proposed synCT method resulted in more closely matched mean HU value to planning CT for soft tissue and lungs. However, the proposed synCT method was found to slightly underestimate the skeleton HUs and to overestimate GI air intensities. Both methods achieved standard deviations of similar magniture for the mean HUs of all investigated tissue types (Supplementary Data D, figure S1).

**Table 3.** Numerical evaluation of the baseline and proposed synCT in terms of structure-based metrics. Dice similarity coefficient (DSC) and Hausdorff distance (HD) were calculated between ground-truth contours (on CT and CBCT) and automatically generated contours (on CT, CBCT and synCT). The mean HU values were calculated within the ground-truth contours (on CT and CBCT).

		Planning CT	CBCT	Baseline synCT	Proposed synCT
**Soft tissues**	**DSC**	0.973 ± 0.007	0.978 ± 0.006	0.971 ± 0.007	0.974 ± 0.007
	**HD**	1.5 ± 0.6	1.4 ± 0.5	2.3 ± 1.0	2.1 ± 1.1
	**Mean HU**	2 ± 11	0 ± 26	24 ± 20	9 ± 20
**Skeleton**	**DSC**	0.896 ± 0.011	0.889 ± 0.022	0.849 ± 0.024	0.862 ± 0.020
	**HD**	1.5 ± 0.8	2.0 ± 2.5	5.9 ± 6.6	3.4 ± 5.2
	**Mean HU**	350 ± 33	359 ± 33	328 ± 27	313 ± 27
**GI air**	**DSC**	0.910 ± 0.055	0.908 ± 0.037	0.846 ± 0.052	0.872 ± 0.053
	**HD**	3.0 ± 4.9	2.7 ± 3.2	9.3 ± 7.2	6.1 ± 6.5
	**Mean HU**	−814 ± 86	−737 ± 93	−775 ± 113	−756 ± 95
**Lungs**	**DSC**	0.956 ± 0.017	0.928 ± 0.034	0.898 ± 0.055	0.898 ± 0.059
	**HD**	2.1 ± 0.9	2.2 ± 0.6	3.9 ± 2.2	3.5 ± 2.0
	**Mean HU**	−526 ± 102	−573 ± 67	−497 ± 74	−547 ± 71

#### 3.3.3. Radiotherapy-specific metrics

**Figure 10.**
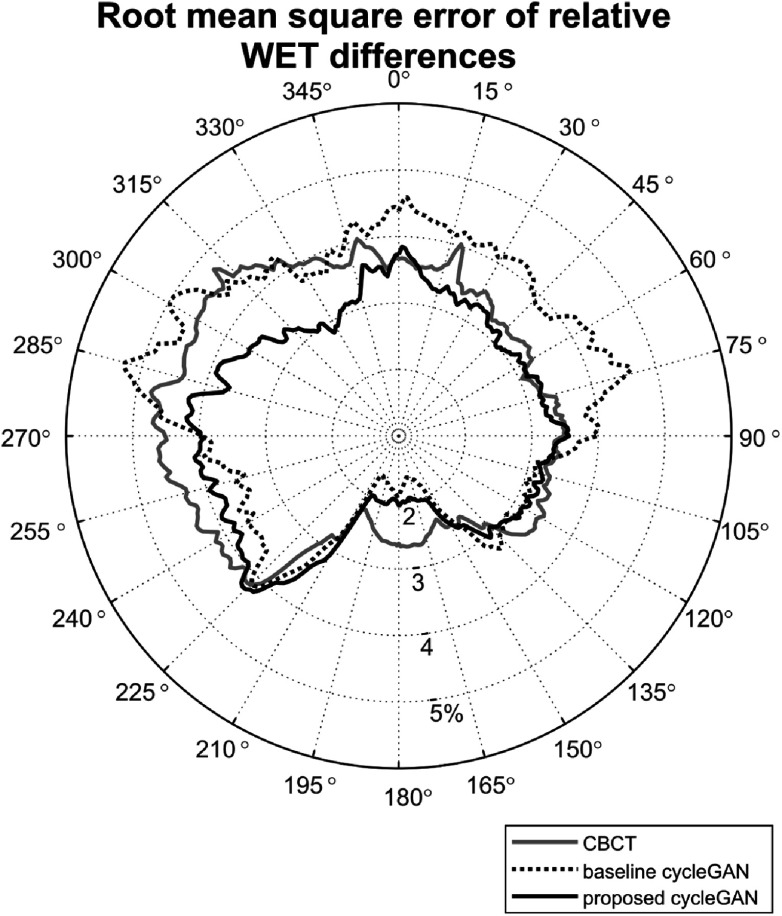
Polar map of the root mean square error of the relative water equivalent thickness (WET) differences between virtual CT (ground-truth) and CBCT and synthetic CT images.

The RMSE in relative WET differences between vCT (ground-truth) and CBCT, baseline synCT and proposed synCT was 3.6 ± 2.6%, 3.7 ± 2.8% and 3.3 ± 2.4%, respectively, when considering all gantry angles and all individual slices. The ${\mathrm{\Delta }}\mathrm{WET}$ for different gantry angles is shown in figure 10. The WET measured for the proposed synCT best matched the vCT WET for anterior and anterior-oblique angles (0° to 90° and 270° to 360°), likely due to its better spatial representation of the GI air pockets. The differences were smaller in the posterior direction, with the baseline synCT being more similar to the vCT in terms of WET for gantry angles between 160° and 270°. This was likely due to these beam angles crossing through higher intensity regions, such as vertebrae and liver and kidneys (in contrast enhanced scans).

## 4. Discussion

We proposed and thoroughly evaluated a novel method for generating synthetic CTs from CBCTs based on cycleGANs. To the best of our knowledge, this is the first study to incorporate structural consistency loss and global residual learning with a weakly paired data approach into CBCT-to-CT synthesis. Our novel smart slice selection framework was shown to facilitate training in diverse populations by allowing one to combine data from multiple patient cohorts in an optimal and efficient strategy. Global residuals learning combined with structural consistency loss helped to improve the structure correspondence between the input and output images, producing synthetic CT images that more closely preserve the structural information of the CBCT.

To the best of our knowledge, Uh *et al* (2021) was the only other study that has also investigated using cycleGANs for CBCT-to-CT synthesis in children and young adults. In their study the networks were trained on abdominal and pelvic datasets using a configuration of cycleGAN very close to what we defined as the baseline method in our study (a key difference was the use of ResNet as generator). The authors followed similar pre-processing steps to what we employed, such as intensity clipping, excluding information external to the body and applying axial normalisation (but only in left-right direction). They reported a mean absolute error of 47 ± 7 HU, excluding regions with GI air, for their best configuration. We report slightly higher differences in our study (55 ± 17 HU). This is likely due to two key differences between our studies which make the results hard to compare directly. First, the CT images we used for training and evaluation included scans both with and without contrast enhancement, which could result in increased pixel intensity values for some organs (for instance liver or kidneys). Second, we did not exclude GI air regions from the analysis, which are the most difficult regions to synthesise. Regarding the integrity of GI air pocket location in the synCT, Uh *et al* (2021) reported a DSC in the range 0.71 – 0.88, compared to 0.82 – 0.92 (average, 0.87 ± 0.05) in our study, which indicates that our method was more successful at preserving GI air pocket location in the synthetic images. In general, their results indicated structural consistency problems like we found for our baseline cycleGAN, such as bones disappearing or pockets of GI air being introduced at wrong locations. Finally, there were also important differences in the methods used to report the quality of the synCTs — our analysis of image quality metrics was more comprehensive but did not explore in as much detail dosimetric aspects.

In the ablation experiment we observed steady improvements in the performance of the framework by introducing all the proposed elements to the cycleGAN framework. The largest quantitative improvement was observed after incorporating the concept of global residuals learning for both generator architectures. Global residuals reframed the problem from generating synthetic images to improving the quality of images (or degrading them in case of the CT-to-CBCT synthesis arm of the cycleGANs). The assumption here is that CBCT represents a corrupted CT image with unwanted artefacts and that the generators can estimate only those artefacts to unveil the underlying artefact-free CT image. Our study demonstrated that this approach was suitable for CBCT to CT synthesis as both imaging modalities share physical acquisition principles.

A limitation of using cycleGANs in medical image synthesis is that the original implementation focused on the translation of images from one domain to another and did not explicitly promote structural consistency between source and synthetic images. As consequence the output images may have fit well into the target domain visually but lacked structural details of the input images. To ensure structural similarity between CBCT and synCT images, our synthesis framework included a structure consistency loss that optimised the LNCC between the input and synthetic images. Although LNCC is commonly used in many medical image analysis applications, to the best of our knowledge its application to promote structure consistency during CBCT-to-CT synthesis had not been proposed before. Alternative approaches to explicitly encourage direct correspondence between source and synthetic images include using similarity measures such as Modality Independent Neighbourhood Descriptor (Shrivastava *et al* 2017), mean absolute error, gradient difference (Wang *et al* 2019, Dai *et al* 2020), correlation coefficients (Ge *et al* 2019a, 2019b) and gradient correlation (Hiasa *et al* 2018a). Chen *et al* (2020) combined mean absolute error loss with structure dissimilarity loss to encourage whole structure wise similarity. Ouyang *et al* (2019) employed a feature-matching technique where a new objective function was specified. In this case the generator encouraged the synthetic images to match the expected value of features on the intermediate layers instead of forcing it on the final output of the discriminator. An alternative approach is to employ some sort of shape-consistency loss, which promotes similarity between annotated features, such as body contours (Ge *et al* 2019a, 2019b), organ sub-volumes (Zhang *et al* 2018, Cai *et al* 2019) or different tissue types (Fang Liu *et al* 2018) between source and synthetic images. This is often employed in conjunction with segmentation networks to facilitate feature identification during the cycleGANS training. Changes to the generator architecture to capture multi-scale information have also been proposed, together with changes to the loss function to generate less blurry images (Lei *et al* 2019). Others have used attention gates (Oktay *et al* 2018) incorporated in the generated architecture to learn structural variations, improving prediction of the image intensities and organ boundaries (Liu *et al* 2020). Some solutions however are only valid when paired data is used for training, as the synthetic image is compared to some sort of ground-truth image. Our proposed approach with LNCC is task dedicated, making use of a well-suited similarity measure known to work well for two image modalities between which a linear relationship can be established. Additionally, the embedding gaussian noise and smoothing of the images helped to enhance global structure preservation. Our results showed that the introduction of the structure consistency loss improved the results for every configuration tested.

To address limitations in data available for disease specific cohorts, we proposed a weakly paired data method for training data selection. Maspero* et al* (2020b) also proposed combining multiple datasets from different anatomical sites to generate a single, generalised network capable of performing on multiple regions. In their study, scans from 33 head and neck, 33 lung and 33 breast subjects, with 15/8/10 split per site were used training validation and testing of a cycleGAN. The reported differences between single-site networks and a combined network trained on a dataset of combined sites were of up to 3 HU (mean value), and no statistical significance was reported. Similarly, Uh *et al* (2021) used data from two patient groups to correct CBCT scans of children and young adults (28 abdominal and 36 pelvic, 64 cases in total). They found that the model trained on the combined dataset significantly outperformed the abdomen and pelvis models in terms of mean absolute HU error of the corrected CBCT from 14 testing patients (47 ± 7 HU versus 51 ± 8 HU). It is possible that the relatively small number of cases per site used for training led to the conclusion that including all the sites in the training results in a better method performance. Moreover, both studies used relatively well-balanced datasets with roughly equal number of cases for each anatomical site included. Our proposed approach, where images were initially registered to a common reference space and then sampled only from a chosen anatomical region is a less naïve technique of combining cases from different anatomical sites. Multiple anatomical regions can be combined in a systematic manner, such that all regions are similarly represented during training. The weakly paired approach improved the performance in the ablation study regardless of the chosen generator architecture and other settings. This demonstrates that it is advantageous to carefully consider presentation strategies of the data to the networks. There is merit into making the training datasets representative, not only larger, as more data did not necessarily lead to better performance.

Training an unsupervised model is a difficult task. We opted to train all models for a fixed number of epochs, while reducing learning rate, stabilizing the networks. Since all methods compared were based on the same generator architecture, this allowed for a reliable comparison of their performance. Early stopping the training, based on a metric calculated on a validation subset, could be an alternative to the training approach we followed. However the choice of the metric (or a combination of multiple metrics) would become another challenge and variable within the framework. Our approach with a fixed number of epochs made it also consistent with other works in the field (Maspero *et al* 2020b, Uh *et al* 2021).

The acquisition of paired CBCT and CT images is not easily feasible. With paired data the synthesis could be framed as a supervised regression problem. Qiu *et al* (2021) performed deformable image registration between planning CTs and CBCTs, which resulted in pseudo paired data and allowed for fully supervised learning. Such an approach is heavily dependent on the quality of the registration between planning CT and CBCT. Achieving accurate registration results may be challenging, particularly in the case of abdominal scans where additional post-processing steps will likely be required to deal with significant bowel gas changes and may still not always be successful. These challenges were demonstrated in our work when generating the virtual CTs for evaluation purposes. While the DIR-based vCT grossly corrected for both structural and intensity differences between pairs of CT and CBCT scans, it was not a perfect representation of a CT-like scan paired to a CBCT. Therefore, in our opinion unsupervised approaches, where paired data are not required, are better suited for the proposed task.

The segmentation-based evaluation allowed to explore in more detail how well different types of tissue are represented in the synCTs. It is important to note that our aim was not to develop a segmentation algorithm per se, but to generate quantitative and automated measures of the structural and intensity quality of the synCT. There were challenges associated with manual and automated segmentation of each tissue type. Segmentation of GI air pockets was associated with uncertainties due to scatter and motion artifacts. It was not always clear from a low quality CBCT where to draw a boundary between gas and tissue. For example, we could observe that the automated GI air segmentation was able to split air regions into individual air pockets, while manual segmentations were more likely to connect them into larger pockets. Furthermore, visual inspection indicated mismatch between the methods in identifying small pockets of gas. Errors in the automatic skeleton segmentation mostly originated from contrast agents being classified as skeleton. Our pre-processing step, where most of contrast agent areas were replaced with water equivalent HU values, contributed to minimise issues in skeleton segmentation. The lung volumes were underrepresented in the testing dataset since only a small fraction of the abdominal scans will contain this tissue type. Motion artifacts at the diaphragm also contributed to making it increasingly challenging to segment the lungs both manually and automatically. DSC and HD metrics were calculated on small sections of the total lung size which may be reflected in the scores. While these uncertainties may impact the results, in our opinion the methodology employed was accurate enough to compare structural similarity between the different synthesis methods.

The analysis of the HUs for individual body tissue types indicated that some challenges remain in terms of generating synthetic CTs with the same intensity information of the matched planning CT. For instance, the mean value of the skeleton in CT was 359 HU, whereas in the proposed synCT it was 313 HU. Intensity mismatch may propagate to dosimetric errors. While our method led to relatively small WET differences (when compared to the baseline synCT) particularly for anterior proton beams, there were no clear advantages for posterior proton beams. These angles could be associated with path lengths that cross the vertebrae and organs such as liver and kidneys (in contrast enhanced scans), where HUs errors were more pronounced. The observed differences could potentially be addressed by applying global histogram normalisations (Zimmerman *et al* 1988, Sandfort *et al* 2019) as a post processing or by introducing additional loss functions within the optimisation framework (Li *et al* 2019a, 2019b, Afifi *et al* 2020).

One of the limitations observed in our study was occasional inconsistency between adjacent slices. Our models were trained with real world CT and CBCT images from a variety of scanners and acquisition settings. CT images used for training were both with and without contrast agent injection, which could lead to inconsistencies where some slices were generated with contrast and others not. This was not entirely unexpected, as our approach was 2D with no explicit adjacent slice consistency enforced. With access to larger datasets, this could be solved by restricting the selection of CT scans used for training and ensuring that only one type of scans was included (with or without contrast). Alternatively, we are considering exploring 3D networks which are expected to improve consistency between adjacent slices but would come with larger memory and computational requirements.

The pre-processing method applied to all images corrected for the presence of elements such as anaesthesia equipment, shunts and lines. Since these elements are very common in the paediatric cohort, we realised during our preliminary studies that the networks learned to spontaneously generate them in the synCT even when they were not present in the source CBCT. We expect that this is more likely to happen when the networks do not enforce structure similarity, so it is likely that this step is not as important to train our proposed network. This should be investigated in the future. Clinically, these elements are avoided by the treatment beams if possible but, if they are inside the treatment volumes, the typical procedure is to override their density for accurate dose calculations. The impact of their incorrect representation in synCTs could be corrected simply by propagating contours from the source CBCT.

The proposed framework could be further improved by finetuning hyperparameters of the optimisation stage (learning rate and its scheduler, optimiser and its parameters, etc), data presentation (data augmentation parameters) and loss calculation (individual loss weights). We decided to keep these fixed on all the experiments after an initial parameter values search so that the results presented for multiple configurations could be more easily and directly comparable.

In the future it would be interesting to investigate the performance of the cycleGAN models in low dose CBCTs. In paediatric radiotherapy lower dose protocols are of interest (Bryce-Atkinson *et al* 2021) but may potentially result in lower quality images and make the learning task even more challenging task. Gao *et al* (2021) investigated different GAN configurations in CBCT-to-CT synthesis in the thorax, noting that the increased imaging artifacts inherent to lower dose CBCT protocols will disturb image translation tasks.

## 5. Conclusions

The proposed framework showed improved quality of synCTs generated from CBCTs when employing strategies to preserve structural consistency and to account for variable field-of-view in the training dataset. The reformulation of the problem from generating synthetic images to refining image quality by applying global residuals only learning led to the biggest improvements. Our study demonstrated the advantages of a thought-through data pre-processing and presentation to the AI method to improve its performance on challenging real-world applications, with scarce and diverse data. A multi-step and multi-layer evaluation allowed us to show that the proposed method resulted in more realistic synCT generation. Further evaluation using metrics of anatomical plausibility and realism, as well as impact on dose calculations, is needed to provide further insight into clinical utility.

## Data Availability

The data cannot be made publicly available upon publication because they are owned by a third party and the terms of use prevent public distribution. The data that support the findings of this study are available upon reasonable request from the authors.
